# *RAD*51 Gene 135G/C polymorphism and the risk of four types of common cancers: a meta-analysis

**DOI:** 10.1186/1746-1596-9-18

**Published:** 2014-01-23

**Authors:** Dan Cheng, Huimin Shi, Kan Zhang, Lingling Yi, Guohua Zhen

**Affiliations:** 1 Department of Respiratory and Critical Care Medicine, Tongji Hospital, Tongji Medical College, Huazhong University of Science and Technology, Wuhan, Hubei, China; 2 Key Laboratory of Respiratory Diseases, Ministry of Health, Wuhan, Hubei, China

**Keywords:** *RAD*51, Single nucleotide polymorphism, Cancer risk, Meta-analysis

## Abstract

**Objectives:**

*RAD*51 gene plays an important role in the pathogenesis of squamous cell carcinoma of the head and neck (SCCHN), colorectal cancer, ovarian cancer and acute leukaemia. A number of studies assessed the association between *RAD51* 135G/C polymorphism and the risk of these cancers in different population. However, the results have been inconclusive. We performed a systematic meta-analysis to evaluate the association between *RAD51* 135G/C polymorphism and the risk of these four types of cancer.

**Methods:**

Pubmed, Cochrane library and Chinese Biomedical Literature Database (CBM) were searched for case-control studies on *RAD*51 135G/C polymorphism and the risk of SCCHN, colorectal cancer, ovarian cancer and acute leukaemia published up to Oct 31, 2013. Odds ratios (ORs) with 95% confidence intervals (CIs) were used to assess the strength of association.

**Results:**

A total of twenty-two published studies, with 6836 cases and 8507 controls were included. Overall, no significant association was found between *RAD51* 135G/C polymorphism and the risk of the four types of cancers (G/G vs. C/C: OR = 0.83, 95% CI: 0.43-1.59, *P* = 0.57). However, there was a significant association between this polymorphism and SCCHN risk in the subgroup analysis by cancer type (G/G vs. C/C: OR = 2.46, 95% CI: 1.08-5.61, *P* = 0.03).

**Conclusion:**

The *RAD*51 135G/C polymorphism was associated with the risk of SCCHN.

**Virtual slides:**

The virtual slide(s) for this article can be found here: http://www.diagnosticpathology.diagnomx.eu/vs/1383180234106945.

## Introduction

Cancer is one of the most common fatal diseases, which results from complex interactions between environmental and genetic factors [1]. More and more studies have focused on the role of gene polymorphism in the aetiology of cancers. Recently, there is growing evidence that single nucleotide polymorphism (SNP) plays an important role in carcinogenesis [2,3]. DNA repair systems have been considered to maintain genomic integrity by counting threats posed by DNA lesions. Deficiency in the DNA repair pathways might make these lesions unrepaired or repaired incorrectly, eventually leading to genome instability or mutations which may contribute directly to cancer.

*RAD51* gene is located on chromosome 15q15.1 in humans [4]. The RAD51 protein encoding by *RAD51* gene is essential for the repair of DNA damage. Growing evidences show that RAD51 has an irreplaceable role in the maintenance of genomic stability and the repair of DNA double-strand breaks [5]. The *RAD51* genetic variations may contribute to the development of cancers [6]. A functional single nucleotide polymorphism, 135G/C (rs1801320), has been identified in the 5′ untranslated region of the *RAD51* gene [7] and has been reported to affect gene transcription activity [8].

Up to now, a variety of molecular epidemiological studies have been conducted to estimate the association between the *RAD51* 135G/C polymorphism and risk of various cancers [9-17], including squamous cell carcinoma of the head and neck [18-21], colorectal cancer [22-25], ovarian cancer [26-28] and acute leukaemia [29-37]. However, the results of previous studies on the association between *RAD51* 135G/C polymorphism and cancer risk have been inconclusive, partially because of the relatively small sample size of most studies. Therefore, we carried out this meta-analysis to evaluate the association between *RAD51* 135G/C polymorphism and risk of the four common types of cancers.

## Methods

### Selection of eligible studies

We conducted a comprehensive search in Pubmed, Cochrane library and Chinese Biomedical Literature Database (CBM), covering all articles published up to Oct 31, 2013, using the following terms: “*RAD51*” AND “polymorphism” AND “(squamous cell carcinoma of the head and neck) OR (colorectal cancer) OR (ovarian cancer) OR (acute leukaemia)”. References of all identified studies and reviews were examined for additional articles.

### Study assessment

Included studies in this meta-analysis met the following criteria: (a) a human case-control study on the association between *RAD51* 135G/C polymorphism and any of the four common cancers; (b) containing available genotype data in cases and controls for estimating an odds ratio (OR) and 95% confidence interval (CI); (c) genotype distributions of control population were consistent with Hardy-Weinberg equilibrium (HWE). The exclusion criteria were: (a) reviews, letters, editorial articles and case reports; (b) studies involving only a case population; (c) research not providing cancer information.

### Data extraction

Two investigators (Cheng and Shi) extracted the data from all of the eligible publications according to the inclusion and exclusion criteria mentioned above. Primary extraction data were reviewed by Zhen, and any disagreement was resolved by discussion among the three authors. From each study, the following information was collected: first author’s name, year of publication, study location, cancer type, sample size, source of control, the genotyping method, the number of genotype frequencies in cases and controls.

### Statistical analysis

For each case-control study, we first examined whether the genotype frequencies in controls were consistent with HWE. ORs and 95% CIs were calculated as a measure of the association between the *RAD51* 135G/C gene polymorphism and risk of the four cancers. The pooled ORs were performed for the homozygote comparison (G/G vs. C/C), heterozygote comparison (G/C vs. C/C), dominant (G/G + G/C vs. C/C) and recessive (G/G vs. G/C + C/C) genetic model comparison, and the significances of the summary ORs were determined by *Z* test, *P* < 0.05 was considered as statistically significant. The chi-square-based *Q*-test was used to assess the statistical heterogeneity among studies, and it was considered significant if *P* < 0.10 [38]. If the *P* value greater than 0.10, indicating the absence of heterogeneity, then a fixed-effects model (the Mantel-Haenszel method) was applied to calculate the summary ORs [39]. Otherwise, the random-effects model (the DerSimonian and Laird method) was used [40]. *I*^2^ was also calculated to test heterogeneity among included studies, with *I*^2^ < 25%, 25-75%, and >75% considered to represent low, moderate and high degree of heterogeneity, respectively [41]. Sensitivity analysis was performed to estimate the stability of the results, each study involved in this meta-analysis was deleted each time to reflect the influence of the individual data set to pooled ORs. Publication bias within the literature was assessed using Begg’s test [42], an asymmetric funnel plot showed a potential publication bias. Egger’s linear regression test (*P* < 0.05 was considered significant publication bias) was also used to evaluate the symmetry of the funnel plot [43]. All of the analyses were carried out with RevMan 5.0.23 (Cochrane Library Software, Oxford, UK) and STATA11.0 (STATA Corporation, College Station, TX, USA).

## Results

### Study characteristics

**Figure 1:**
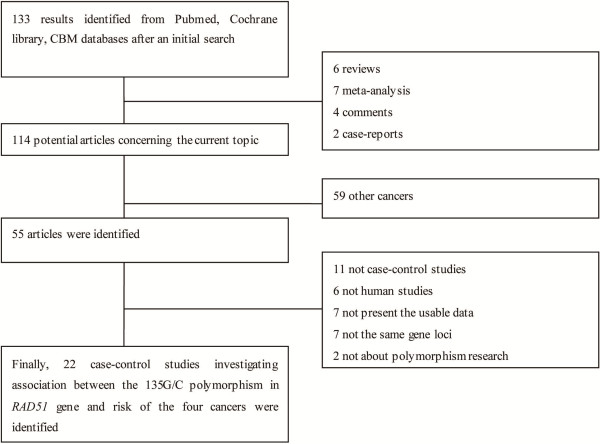
Flow diagram of study selection.

A total of 133 related publications were identified, of which 19 studies were not accepted since they were not full articles (6 reviews, 7 meta-analysis, 4 comments, 2 case-reports). Fifty-nine articles were not about the above four cancers, 33 publications were excluded because they did not meet the inclusion criteria (11 not case-control studies, 6 not human studies, 7 not present the usable data, 7 not the gene loci, 2 not about polymorphism research). Finally, 22 studies including 6836 cases and 8507 controls were included in this meta-analysis (Figure 1).

The main characteristics of these 22 included studies are summarized in Table 1. There were 14 studies from European countries, 3 studies from Asian countries, 3 studies from American countries, 1 study from Australia and 1 study from Africa. In addition, 9 articles were population-based and 13 articles were hospital-based. The number of publications on SCCHN, colorectal, ovarian cancer and acute leukaemia were 4, 4, 5, and 9, respectively. The diagnosis of most of the cases was based on pathology. Healthy subjects matched for age and sex were used as controls. Polymerase chain reaction (PCR) or restriction fragment length polymorphism(RFLP) were performed as genotyping methods. The genotype distributions and HWE examination results were shown in Table 2.

**Table 1:** Characteristics of 22 published studies included in this meta-analysis

**First author**	**Year**	**Study location**	**Cancer type**	**Sample size**	**Source of controls**	**Genotyping methods**
1. Lu JC	2006	USA	SCCHN	716/719	HCC	PCR-RFLP
2. Gil J	2011	Poland	CC	133/100	HCC	PCR-RFLP
3. Webb PM	2005	Australia	OC	548/335	PCC	PCR
4. Gresner P	2012	Poland	SCCHN	81/111	PCC	PCR
5. Seedhouse C	2004	UK	AL	267/186	PCC	PCR
6. Jawad M	2006	UK	AL	267/186	PCC	PCR
7. Krupa R	2011	Poland	CC	100/100	HCC	PCR
8. Sliwinski T	2010	Poland	SCCHN	288/353	HCC	PCR-RFLP
9. Hamdy MS	2011	Egypt	AL	50/30	HCC	PCR-RFLP
10. Liu L	2011	China	AL	625/704	HCC	PCR
11. Romanowicz-MakowskaH	2012	Poland	CC	320/320	HCC	PCR
12. WerbouckJ	2008	Belgium	SCCHN	152/157	HCC	PCR
13. Romanowicz-MakowskaH	2011	Poland	OC	120/120	HCC	PCR
14. Bhatla D	2008	USA	AL	452/646	PCC	PCR
15. Voso MT	2007	Italy	AL	160/161	HCC	PCR-RFLP
16. Mucha B	2012	Poland	CC	200/200	HCC	PCR-RFLP
17. Zhang ZQ	2009	China	AL	166/458	HCC	PCR-RFLP
18. Auranen A (UK)	2005	UK	OC	729/847	PCC	PCR
18. Auranen A (USA)	2005	USA	OC	326/419	PCC	PCR
18. Auranen A (Danish)	2005	Denmark	OC	278/699	PCC	PCR
21. Yang L	2011	China	AL	379/704	HCC	PCR
22. Rollinson S	2006	UK	AL	479/952	PCC	PCR

SCCHN: squamous cell carcinoma of the head and neck; CC: colorectal cancer; OC: ovarian cancer; AL: acute leukaemia; HCC: hospital-based case-control; PCC: population-based case-control; PCR: polymerase chain reaction; RFLP: restriction fragment length polymorphism.

**Table 2:** Distribution of RAD51 genotype and allele among cancer patients and controls

**First author**	**Year**	**Case**	**GC**	**CC**	**Control**	**GC**	**CC**	**Case**	**C**	**Control**	**C**	**HWE**
		**GG**			**GG**			**G**		**G**		
1. Lu JC	2006	624	91	1	622	96	1	1339	93	1340	98	0.17
2. Gil J	2011	100	29	4	73	27	0	229	37	173	27	0.19
3. Webb PM	2005	457	85	4	971	145	10	999	93	2087	165	0.08
4. Gresner P	2012	67	13	1	71	14	2	147	15	156	18	0.22
5. Seedhouse C	2004	210	44	3	166	18	2	464	50	350	22	0.08
6. Jawad M	2006	210	44	3	166	18	2	464	50	350	22	0.08
7. Krupa R	2011	61	36	3	36	35	29	158	42	107	93	0.003
8. Sliwinski T	2010	138	145	5	258	64	32	421	155	580	128	0.28
9. Hamdy MS	2011	39	9	2	26	3	1	87	13	55	5	0.06
10. Liu L	2011	72	25	8	511	175	18	169	25	1197	211	0.52
11. Romanowicz- MakowskaH	2012	51	56	213	91	164	65	158	482	346	294	0.57
12. Werbouck J	2008	136	15	1	134	23	0	287	17	291	23	0.32
13. Romanowicz-MakowskaH	2011	13	15	92	33	69	18	41	199	135	105	0.07
14. Bhatla D	2008	374	73	5	555	85	6	821	83	1195	97	0.18
15. Voso MT	2007	125	33	2	142	18	1	283	37	302	20	0.61
16. Mucha B	2012	161	34	5	157	37	6	356	44	351	49	0.05
17. Zhang ZQ	2009	117	47	2	315	123	20	281	51	753	163	0.08
18. Auranen A (Danish)	2005	241	36	1	616	78	5	518	38	1310	88	0.15
18. Auranen A (UK)	2005	642	84	3	745	100	2	1368	90	1590	104	0.48
18. Auranen A (USA)	2005	270	52	4	357	61	1	592	60	775	63	0.34
21. Yang L	2011	268	101	10	511	175	18	637	121	1197	211	0.52
22. Rollinson S	2006	431	34	1	817	115	4	896	36	1749	123	0.98

HWE: *P* value for Hardy-Weinberg equilibrium for *RAD51* 135G/C polymorphism among controls.

### Quantitative synthesis

**Figure 2:**
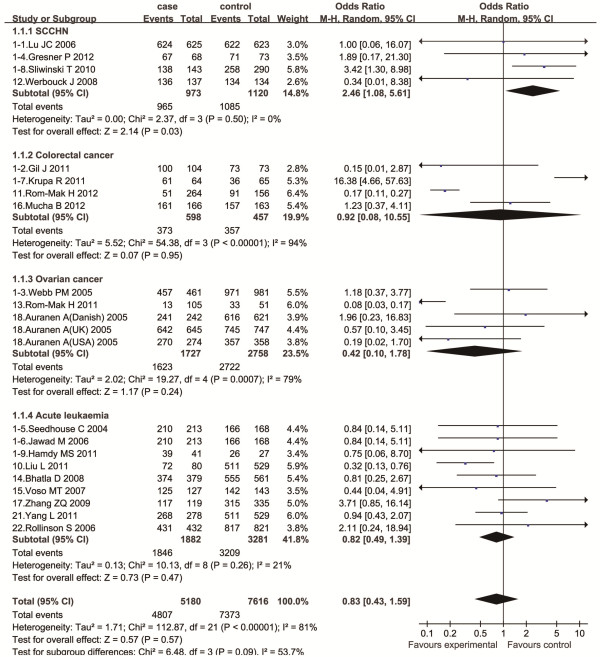
**The association between *RAD51 *** **135G/C polymorphism and the four common cancers risk in the subgroup analysis by cancer type (GG vs. CC).**

The evaluation of association between *RAD*51 135G/C gene polymorphism and the risk of the four types of cancers was summarized in Table 3. Overall, no significant association was found between *RAD*51 135G/C gene polymorphism and the risk of the four cancers (G/G vs. C/C: OR = 0.83, 95%CI = 0.43-1.59, *P* = 0.57; G/C vs. C/C: OR = 0.90, 95%CI = 0.39-2.08, *P* = 0.81; G/G + G/C vs. C/C: OR = 0.82, 95%CI = 0.39-1.73, *P* = 0.60; G/G vs. G/C + C/C: OR = 0.84, 95%CI = 0.69-1.02, *P* = 0.08). However, in the subgroup analysis by cancer type, there was a significant association between this polymorphism and SCCHN under homozygote comparison (G/G vs. C/C: OR = 2.46, 95%CI = 1.08-5.61; *P* = 0.03) (Figure 2). There was no significant association between this polymorphism and the risk of other three cancers under all comparisons. In the subgroup analyses by ethnicity or source of controls, no significant association was found in different genetic models.

**Table 3:** Total and stratified analysis of the RAD51 135G/C polymorphism on risk of the four cancers

**Variables**	**No.**^**a**^	**Case/Control**	**GG vs. CC**			**GC vs. CC**			**GG+GC vs. CC**			**GG vs. GC+CC**		
			**OR(95% CI)**	**P**^**b**^	**P**	**OR(95% CI)**	**P**^**b**^	**P**	**OR(95% CI)**	**P**^**b**^	**P**	**OR(95% CI)**	**P**^**b**^	**P**
**Total cancer types**	22	6836/8507	0.83(0.43–1.59)	0.00^c^	0.57	0.90(0.39–2.08)	0.00^c^	0.81	0.82(0.39–1.73)	0.00^c^	0.60	0.84(0.69–1.02)	0.00^c^	0.08
SCCHN	4	1237/1340	2.46(1.08–5.61)	0.50	0.03	2.20(0.30–16.22)	0.02	0.44	2.50(0.76–8.28)	0.25	0.13	0.84(0.40–1.75)	0.00^c^	0.64
CC	4	753/720	0.92(0.08–10.55)	0.00^c^	0.95	0.65(0.05–8.08)	0.00^c^	0.74	0.79(0.06–10.16)	0.00^c^	0.85	1.12(0.53–2.35)	0.00^c^	0.77
OC	5	2001/2420	0.42(0.10–1.78)	0.0007	0.24	0.41(0.06–2.67)	0.00^c^	0.35	0.40(0.07–2.18)	0.00^c^	0.29	0.80(0.62–1.03)	0.07	0.09
AL	9	2845/4027	0.82(0.49–1.39)	0.26	0.47	1.00(0.59–2.08)	0.29	0.99	0.85(0.50–1.44)	0.25	0.60	0.82(0.63–1.07)	0.002	0.14
**Source of controls**														
HCC	13	3409/4126	0.77(0.30–1.98)	0.00^c^	0.58	0.79(0.24–2.60)	0.00^c^	0.70	0.74(0.26–2.13)	0.00^c^	0.58	0.82(0.60–1.12)	0.00^c^	0.20
PCC	9	3427/4381	0.93(0.53–1.62)	0.87	0.79	1.14(0.64–2.02)	0.87	0.66	0.95(0.54–1.67)	0.88	0.87	0.88(0.71–1.09)	0.01	0.25
**Ethnicity**														
Asian	3	1170/1866	0.92(0.27–3.19)	0.01	0.90	0.98(0.28–3.40)	0.01	0.97	0.94(0.27–3.26)	0.009	0.92	0.94(0.77–1.14)	0.64	0.52
Caucasian	18	5616/6611	0.80(0.36–1.79)	0.00^c^	0.59	0.86(0.31–2.38)	0.00^c^	0.77	0.79(0.32–1.95)	0.00^c^	0.61	0.83(0.65–1.05)	0.00^c^	0.13
African	1	50/30	0.75(0.06–8.70)		0.82	1.50(0.10–23.07)		0.77	0.83(0.07–9.54)		0.88	0.55(0.16–1.90)		0.34

^a^Number of studies.

^b^P value of Q-test for heterogeneity test.

^c^Fixed-effects model was used when P value for heterogeneity test <0.10, otherwise, random-effects model was used.

SCCHN: squamous cell carcinoma of the head and neck; CC: colorectal cancer; OC: ovarian cancer; AL: acute leukaemia.

### Test of heterogeneity

For the comprehensive analysis, the *I*^2^ showed a stable variation under all comparisons (G/G vs. C/C: *P* < 0.00001, *I*^2^ = 81%; G/C vs. C/C: *P* < 0.00001, *I*^2^ = 89%; G/G + G/C vs. C/C: *P* < 0.00001, *I*^2^ = 88%; G/G vs. G/C + C/C: *P* < 0.00001, *I*^2^ = 78%). In the subgroup analyses of SCHNN and acute leukaemia, the *I*^2^ showed a low or moderate variation under all comparisons. In the subgroup analyses of colorectal cancer and ovarian cancer, under most comparisons, the moderate heterogeneity was detected. For source of controls, there was no significant heterogeneity under all comparisons of population-based case-control (PCC), except for heterozygous and dominant model comparisons (G/C vs. C/C: *P* < 0.00001, *I*^2^ = 93%; G/G + G/C vs. C/C: *P* < 0.00001, *I*^2^ = 92%) in hospital-based case-control (HCC). *P* value for heterogeneity was not significant under all comparisons in the subgroup analyses of Asian population, but in Caucasian group, there were high degree heterogeneity under heterozygous and dominant model comparisons (G/C vs. C/C: *P* < 0.00001, *I*^2^ = 90%; G/G + G/C vs. C/C: *P* < 0.00001, *I*^2^ = 89%).

### Sensitivity analysis

Sensitivity analyses were performed to assess the stability of the results in this meta-analysis. Statistically similar data were obtained after sequentially excluding each study, indicating that our results were statistically reliable.

### Publication bias

**Figure 3:**
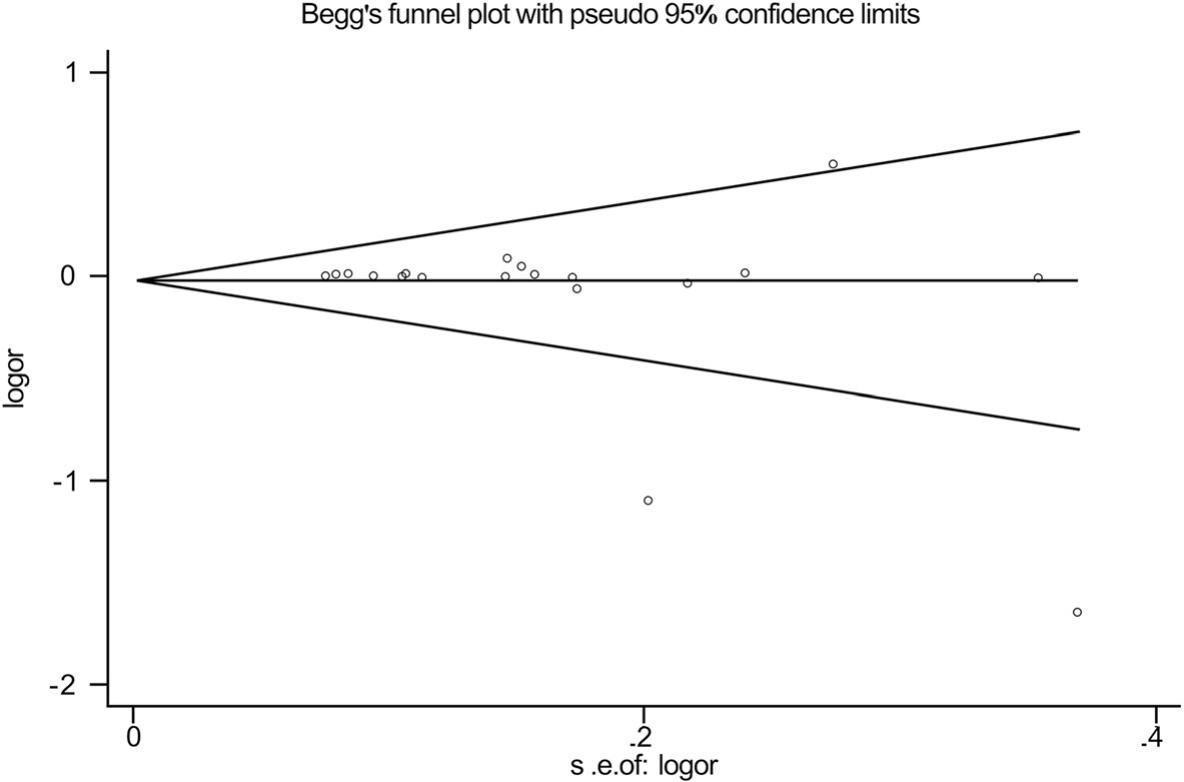
**Begg’s funnel plot for publication bias in selection of studies on *RAD51 *** **135G/C polymorphism (GG vs. CC; *P *** **for bias = 0.248).**

Begg’s funnel plot and Egger’s test were used to assess the publication bias of included studies. Publication bias was not observed in Begg’s funnel plot. The shape of the funnel plots showed to be symmetrical (G/G vs. C/C) and the Egger’s test did not show any evidence of publication bias (*P* = 0.248 for G/G vs. C/C) (Figure 3). These data indicate that there is no significant publication bias in this meta-analysis.

## Discussion

The RAD51 protein encoding by *RAD51* gene is essential for the repair of DNA damage. A number of original studies have reported the association between *RAD51* 135G/C polymorphism and the risk of cancer with inconclusive results, These inconsistent results are possibly because of a small effect of the polymorphism on cancers risk or the relatively low statistical power of the published studies. To better understanding of this association,a meta-analysis, which potentially investigates a large number of individuals and could estimate the effect of a genetic factor on the risk of cancers, was needed to provid a quantitative approach for combining the results of various studies with the same topic, and for estimating and explaining their diversity [44,45]. We performed a meta-analysis including 6836 cases and 8507 controls from 22 case-control studies to evaluate the association between *RAD51* 135G/C polymorphism and risk of SCCHN, colorectal cancer, ovarian cancer and acute leukaemia.

The overall population analysis showed no significant association between *RAD51* 135G/C polymorphism and risk of SCCHN, colorectal cancer, ovarian cancer and acute leukaemia in any genetic model. However, in the subgroup analysis by cancer type, we found that the 135G/C polymorphism of the *RAD51* gene was associated with a significantly increased SCCHN risk. There was an aggregated OR of 2.46 (95% CI = 1.08-5.61) for increased SCCHN susceptibility under homozygote comparison. This indicates that the *RAD51* 135G/C polymorphism may contribute to pathogenesis of SCCHN. GG genotype has been reported to enhance *RAD51* gene transcription activity [8], individuals with GG genotype may be more likely to develop SCCHN than those with CC or GC genotype. No associations were found between this polymorphism and the risk of colorectal cancer, ovarian cancer and acute leukaemia, which was consistent with previous reports [22,23,27-29,32,36].

Heterogeneity is one of the important issues in performing a meta-analysis. In the present meta-analysis, heterogeneity was found in almost all comparisons. Using random-effect models and the stratified analyses by cancer type, sources of control and ethnicity, the heterogeneity was significantly decreased in most of the comparisons. The sensitivity analysis did not alter the results of our meta-analysis, indicating the results are stable. Meanwhile, the publication bias for the association between *RAD51* 135G/C polymorphism and the risk of the four types of cancers were not detected.

The present meta-analysis has some limitations. First, the control subjects were not uniformly defined. Selection bias and classification bias were possible because the included controls may have other different risks of developing cancers. Second, in the subgroup analyses, the sample sizes of Asian and African population were relatively small, not having enough statistical power to explore the real association. Third, cancer is a multi-factorial disease, our meta-analysis was based on unadjusted estimates.

In conclusion, the GG genotype of *RAD51* 135G/C was associated with a significantly increased risk of SCCHN. However, there was no significant association between this polymorphism and colorectal cancer, ovarian cancer or acute leukaemia susceptibility.

## Competing interest

The authors have declared that no competing interests exist.

## Authors' contributions

DC performed the literature search, data extraction, statistical analysis and drafted the manuscript. HS, KZ and LY participated in data extraction. GZ supervised the literature search, data extraction, statistical analysis and drafted the manuscript. All authors read and approved the final manuscript.
